# Detection of early metaphyseal changes in a piglet model of Legg-Calvé-Perthes disease using quantitative mapping of MRI relaxation times

**DOI:** 10.1002/jor.25904

**Published:** 2024-05-26

**Authors:** Erick O. Buko, Alexandra R. Armstrong, Jennifer C. Laine, Ferenc Tóth, Casey P. Johnson

**Affiliations:** 1 Department of Veterinary Clinical Sciences, University of Minnesota, St. Paul, Minnesota, USA; 2 Center for Magnetic Resonance Research, University of Minnesota, Minneapolis, Minnesota, USA; 3 Gillette Children's Specialty Healthcare, St. Paul, Minnesota, USA; 4 Department of Orthopedic Surgery, University of Minnesota, Minneapolis, Minnesota, USA

**Keywords:** growth plate, Legg-Calvé-Perthes disease (LCPD), magnetic resonance imaging, metaphysis, spongiosa

## Abstract

Legg-Calvé-Perthes disease (LCPD) is a childhood hip disorder characterized by ischemic injury to the epiphysis of the femoral head, but changes to the metaphysis have also been implicated in its pathogenesis. Quantitative magnetic resonance imaging (MRI) relaxation time mapping techniques are potentially useful to detect injury in LCPD, but studies to date have focused on the epiphysis. The purpose of this study was to assess whether T2, T1ρ, adiabatic T1ρ, and adiabatic T2ρ relaxation times can detect early metaphyseal changes in an LCPD piglet model. Complete epiphyseal ischemia of one femoral head was surgically induced and confirmed using contrast-enhanced MRI in *n* = 10 6-week-old piglets; the contralateral side was unoperated. The bilateral hips were imaged 1 week after surgery in vivo at 3T MRI using relaxation time mapping and contrast-enhanced MRI. Relaxation times and thicknesses of the metaphyseal primary and secondary spongiosa were measured and compared between the ischemic and contralateral-control femoral heads using paired *t*-tests. In the ischemic femoral heads, T2 relaxation times were significantly increased in the primary spongiosa (6.7 ± 9.8 ms, *p* = 0.029), and T2, T1ρ, adiabatic T1ρ, and adiabatic T2ρ relaxation times were significantly decreased in the secondary spongiosa (respectively: −13.3 ± 9.3 ms, *p* = 0.013; −32 ± 23 ms, *p* < 0.001; −43 ± 41 ms, *p* = 0.009; and −39 ± 13 ms, *p* < 0.001). The secondary spongiosa thickness was also significantly decreased in the ischemic femoral heads (*p* < 0.001). In conclusion, T2, T1ρ, adiabatic T1ρ, and adiabatic T2ρ relaxation time mapping techniques can detect early changes in the metaphysis following ischemic injury to the epiphysis of the femoral head in a piglet model of LCPD.

## 1 | INTRODUCTION

Legg-Calvé-Perthes disease (LCPD) is a childhood form of osteonecrosis of the femoral head caused by interruption of its vascular supply.^1^ Ischemic injury disrupts the normal development of the femoral head, which can lead to permanent hip deformity and premature onset of osteoarthritis. Femoral head changes in LCPD are mostly seen in the epiphysis (i.e., the bone and bone marrow i.e., proximal to the growth plate). In some children, involvement of the metaphysis (i.e., the bone and bone marrow distal to the growth plate) is also apparent, as evidenced by histological alterations,^2^ radiographic changes,^3-5^ discrete areas of increased T2-weighted magnetic resonance imaging (MRI) signal,^6-8^ and diffusely increased diffusion-weighted MRI signal.^9-14^ These metaphyseal changes have been associated with an unfavorable prognosis^3-5,13,14^ and may relate to transphyseal reperfusion of the epiphysis of the femoral head, which can cause growth plate arrest and a poor clinical outcome.^15-17^

Studies of a piglet model of LCPD suggest that metaphyseal changes begin to occur in the early, avascular stage of LCPD. Disruption of the growth plate associated with ischemic injury to the epiphysis leads to thinning of the metaphyseal primary spongiosa in the piglet model by 1 week after the onset of complete epiphyseal ischemia.^18^ The metaphyseal spongiosa is the region of the metaphysis adjacent to the growth plate where conversion of calcified cartilage into mature metaphyseal bone takes place; it includes the primary spongiosa (i.e., the site for mineralization) and secondary spongiosa (i.e., bone remodeling zone) subregions.^19,20^ These early metaphyseal changes may contribute to findings of shortening of the femoral neck and development of radiographic and histological changes in the metaphysis at later disease stages that are similar to those seen in children with LCPD.^21^ However, no medical imaging methods have been identified that are sensitive in detecting these early changes to the metaphyseal spongiosa following ischemic injury to the femoral epiphysis.

MRI relaxation time mapping techniques may be advantageous for imaging the metaphysis in children with LCPD, including very early changes to the metaphyseal spongiosa. Compared to other imaging approaches (radiographs, conventional T2-weighted MRI, and diffusion-weighted MRI), relaxation time mapping techniques provide quantitative information with high spatial resolution and sensitivity to subtle changes to the bone marrow and bone. Quantitative mappings of T2 and T1ρ relaxation times, and adiabatic variants of these methods such as adiabatic T1ρ (aT1ρ) and adiabatic T2ρ (aT2ρ), have been shown to be sensitive in detecting ischemic injury to the epiphysis of the femoral head in studies using the LCPD piglet model.^22-25^ Specifically, these methods are sensitive to the composition of the bone marrow and bone and to the swelling and breakdown of cells that occurs with necrosis.^22,23^ While the prior studies in the LCPD piglet model did not detect relaxation time changes in a large metaphyseal region following onset of femoral head ischemia,^22-25^ the metaphyseal spongiosa region warrants closer investigation. It has previously been noted that T1ρ relaxation times may be particularly sensitive to compositional changes in this region.^23^

The purpose of this study was to determine if T2, T1ρ, aT1ρ, and aT2ρ relaxation times are sensitive in detecting early changes to the metaphyseal spongiosa following ischemic injury to the epiphysis of the femoral head in a piglet model of LCPD. We hypothesized that relaxation times are altered in the metaphyseal primary and secondary spongiosa in ischemic versus contralateral-control femoral heads 1 week after the onset of ischemia.

## 2 | MATERIALS AND METHODS

### 2.1 | Animal model

The University of Minnesota's Institutional Animal Care and Use Committee approved this study. Eleven Yorkshire piglets (7 male, 4 female) were acquired from a commercial provider (Manthei Hog Farm, LLC) and housed in pairs at the University of Minnesota's Research Animal Resources facilities. At 6 weeks of age (mean weight = 10.6±1.8kg, range = 8.1–14.5 kg), piglets underwent surgery to induce complete (100%) ischemia of the epiphysis of the femoral head by placement of a ligature around the femoral neck and transection of the ligamentum teres.^26,27^ The contralateral femoral head remained unoperated and served as a normal, paired control. One week following surgery, each piglet underwent in vivo bilateral hip imaging at 3 T MRI. The 1-week time point was chosen because it corresponds to the early, avascular stage of the LCPD that precedes the revascularization stage (which can initiate by 2 weeks postoperatively in the LCPD piglet model).^27^ By this time, the femoral head is completely necrotic and changes in the metaphysis have started to develop.^18^ During the surgical and MRI procedures, piglets were premedicated using intramuscular administration of either a combination of midazolam (10 mg/kg) and buprenorphine (10 μg/kg) or a combination of telazol (4.0 mg/kg) and xylazine (2.0 mg/kg). Anesthesia was induced by intravenous administration of either propofol (2.0–6.0mg/kg) or both ketamine (5.0 mg/kg) and propofol (2.0–6.0 mg/kg) and maintained by insufflation of isoflurane (1.0%–5.0%) vaporized in oxygen. Post-operative pain control was provided by oral administration of carprofen (2.0–3.0 mg/kg) once or twice daily for 3 days. Three of the 11 piglets were euthanized immediately after MRI with an intravenous injection of potassium chloride (75–150 mg/kg) or sodium pentobarbital (100 mg/kg), and their femoral heads were collected for histological assessment. The remaining eight piglets were allowed to recover from anesthesia for use in other studies, and thus histological assessment was not possible in these animals. An additional four pairs of femoral head specimens from four different piglets that were studied using the identical procedures but for which corresponding MRI data were not acquired were also available for supplemental histological assessments.

### 2.2 | In vivo 3T MRI

One week following surgery, piglets were imaged using a clinical 3 T MRI scanner (MAGNETOM Prisma; Siemens Healthcare) using two vendor-provided four-channel flex receiver arrays. Imaging sequences acquired included: (i) quantitative relaxation time mappings (T2, T1ρ, aT1ρ, and aT2ρ) using a 2D magnetization-prepared turbo spin echo (TSE) sequence; and (ii) subtraction CE-MRI using a 2D TSE sequence acquired before and after intravenous administration of gadolinium contrast material (0.2 mmol ProHance; Bracco Diagnostics). The subtracted CE-MRI image was used to confirm complete induction of femoral head ischemia by lack of signal enhancement in the epiphysis of the operated femoral head. Imaging parameters are listed in Table 1.

### 2.3 | Histology

Seven pairs of femoral heads were available for analysis: three from the 11 piglets of this study, and four from a separate group of piglets that were studied using the same methodologies but without corresponding MRI data. Femoral head specimens were fixed in 10% neutral buffered formalin, decalcified in 10% ethylenediaminetetraacetic acid, processed and embedded in paraffin, and sectioned at 5 μm thickness. Hematoxylin and eosin (H&E) staining was performed according to standard protocols. A board-certified veterinary pathologist performed a qualitative histological assessment of the H&E-stained sections of the femoral heads.

### 2.4 | Image and data analysis

**FIGURE 1:**
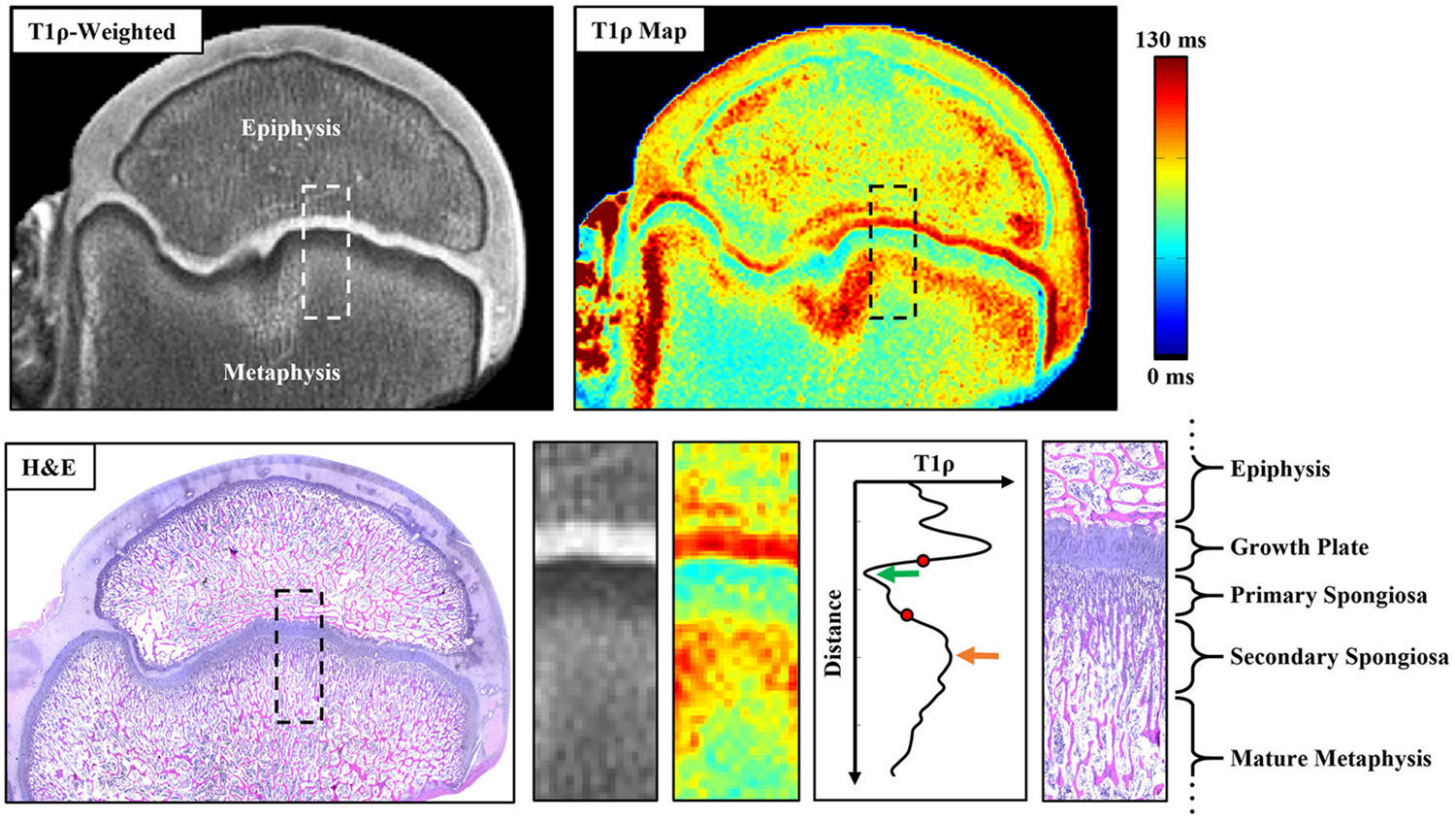
Subregions of the metaphysis in a femoral head specimen from a 6-week-old piglet. The T1ρ-weighted image and corresponding T1ρ relaxation time map of the specimen were acquired at 9.4 T MRI, and a corresponding H&E-stained histological section is shown.^23^ Magnifications of the boxed regions are shown, as well as the signal profile for the corresponding T1ρ map region (with symbols matching those described in Figure 2). The epiphysis and metaphysis are divided by the hyperintense growth plate (which has relative long T1ρ relaxation times). Two distinct subregions of the metaphyseal spongiosa are visible: the hypointense primary spongiosa and the hyperintense secondary spongiosa (which respectively have relatively short and long T1ρ relaxation times). The more distal, mature metaphysis has shorter T1ρ relaxation times than the secondary spongiosa.

Figure 1 illustrates the appearance of the growth plate and metaphysis in a femoral head specimen from a 6-week-old piglet (from a prior study) imaged using high-resolution 9.4 T MRI and histology.^23^ In this case, the histological section was spatially aligned to the ex vivo MRI scan using anatomical landmarks. Based on prior literature of the appearance of MRI signal intensities of the growth plate and metaphysis and histological characterization of the zones of the metaphysis,^19,20,28-30^ we defined the following regions of interest for the current study using Figure 1 as an illustrative example. The *growth plate* was defined as the cartilaginous region between the epiphysis and metaphysis, which is hyperintense on T2- and T1ρ-weighted MRI. The *primary spongiosa* was defined as the subregion of the metaphysis just distal to the growth plate consisting of calcified cartilage and newly formed bone, which is hypointense on T2- and T1ρ-weighted images. This region is sometimes referred to as the “zone of provisional calcification.” The *secondary spongiosa* was defined as the subregion of the metaphysis just distal to the primary spongiosa consisting of immature, woven bone undergoing remodeling and intermixed with vasculature and low cellularity marrow, which is hyperintense on T2- and T1ρ-weighted images relative to both the primary spongiosa and the mature metaphysis. The *mature metaphysis* was defined as the remainder of the metaphysis distal to the secondary spongiosa, which consists of mature trabecular bone and marrow with higher cellularity.

T2, T1ρ, aT1ρ, and aT2ρ relaxation time maps were each generated by fitting a mono-exponential signal decay model on a pixel basis to the series magnetization-prepared TSE images using MATLAB (MathWorks). The relaxation time maps and weighted images were interpolated three-fold to increase the pixel resolution to 0.17mm for the analysis. Rather than defining spatial regions of interest (ROIs) on the relaxation time maps, we instead used a line profile analysis to account for potential variability in the location of the regions between the ischemic and control femoral heads as well as potential differences between piglets. The line profiles were used to measure both the relaxation times within the metaphysis subregions (primary spongiosa, secondary spongiosa, and mature metaphysis) and the thicknesses of the subregions (primary spongiosa, secondary spongiosa, and total metaphyseal spongiosa).

**FIGURE 2:**
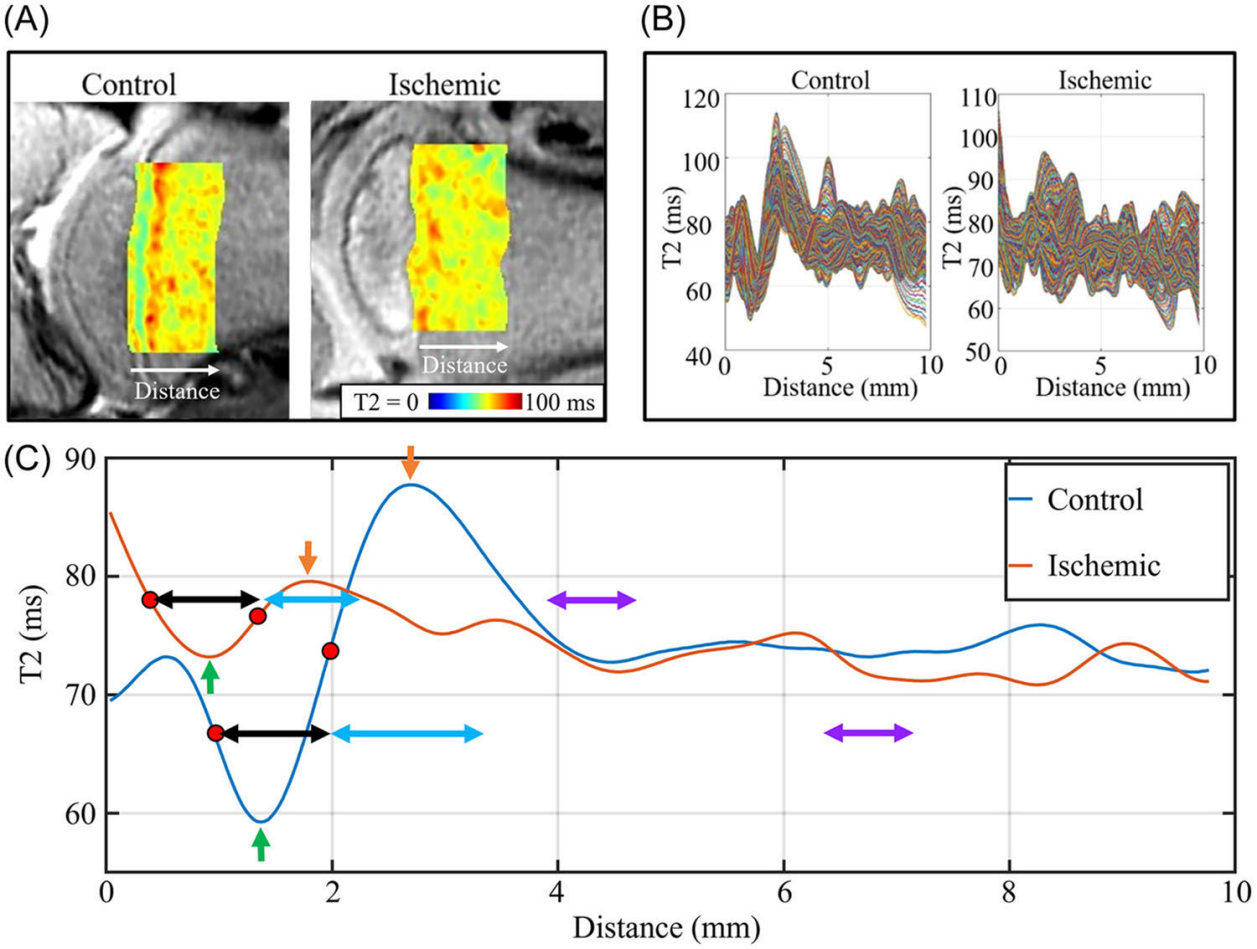
Metaphysis quantitative analysis procedure and definitions. (A) T2 maps within a region of interest (ROI) overlaid on a corresponding T2-weighted images for a pair of control and ischemic femoral heads (note that the images are rotated 90° relative to those shown Figure 1). The ROI defined the region from which line profiles were measured, extending from the proximal edge of the growth plate to a distance of 10 mm into the metaphysis. (B) Plots of the individual line profiles corresponding to each row of the T2 map ROIs shown in (A) from the growth plate (0 mm) into the metaphysis (10 mm). (C) Plots of the average of all line profiles shown in (B) for the control (blue line) and ischemic (orange line) femoral heads. The first minimum represents the primary spongiosa (green arrows), and the first maximum following the primary spongiosa represents the secondary spongiosa (orange arrows). The distance between the two inflection points (red dots) on either side of the primary spongiosa is defined as the primary spongiosa thickness (black arrows). Twice the distance between the inflection point separating the primary and secondary spongiosa and the location of secondary spongiosa is defined as the secondary spongiosa thickness (blue arrows). The total metaphyseal spongiosa thickness is defined as the sum of primary and secondary spongiosa thicknesses. Relaxation times for the mature metaphysis were sampled over an interval of 0.7 mm (purple arrows) located at four times the distance between the primary and secondary spongiosa away from the primary spongiosa.

For each femoral head, the beginnings of the line profiles were traced along the proximal boundary of the growth plate on a magnetization-prepared T2-weighted image, and each line profile extended 10 mm (± 0.5 mm) distally into the metaphysis (Figure 2A). The relaxation times for a given quantitative map were then plotted along each of these line profiles, resulting in a series of plots of relaxation time versus spatial location (Figure 2B). The number of line profiles per femoral head was variable (range = 60–100 lines), but these line profiles were then averaged to get a single representative relaxation time versus distance plot for each femoral head (Figure 2C). Specifically, the series of relaxation time values at a given spatial location were averaged together using the Gaussian mean to represent the average relaxation value at a given depth while also removing the effects of outliers. This average line profile, which is shown in Figure 2C for both the ischemic and contralateral-control femoral heads, had a characteristic relaxation time signal variation that is consistent with the relaxation times differences shown in Figure 1: relaxation times were longer in the growth plate than in the primary spongiosa, shorter in the primary spongiosa than in the secondary spongiosa, and longer in the secondary spongiosa than in the mature metaphysis.

The first minimum point along the average line profile was taken as the relaxation time value and spatial location of the primary spongiosa (Figure 2C, green arrow). The first maximum point following the primary spongiosa was taken as the relaxation time value and location of the secondary spongiosa (Figure 2C, orange arrow). The relaxation time value of the mature metaphysis was taken as the average value across an interval of 0.7 mm (purple arrow) at four times the distance between the locations of the primary and secondary spongiosa. The remaining portion of the average line profile extending beyond the mature metaphysis measurement region (typically 3 to 5 mm) was not used.

The “primary spongiosa thickness” was defined as the distance between the two inflection points on either side of the primary spongiosa's location (Figure 2C, black arrow). The “secondary spongiosa thickness” was defined as twice the distance between the inflection point at the boundary of the primary and secondary spongiosa and the location of the secondary spongiosa (Figure 2C, blue arrow). The total metaphyseal spongiosa thickness was defined as the sum of the primary and secondary spongiosa thicknesses.

These six quantitative measurements (primary spongiosa relaxation time, secondary spongiosa relaxation time, mature metaphysis relaxation time, primary spongiosa thickness, secondary spongiosa thickness, and total metaphyseal spongiosa thickness) were then compared between the pairs of ischemic and contralateral-control femoral heads for each relaxation time map (T2, T1ρ, aT1ρ, and aT2ρ) using paired *t*-tests with a significance threshold set to *p* < 0.05. The pairwise comparisons remove variations between piglets (e.g., size differences) as a potential confounder. We also assessed each measurement's effect size (Cohen's *d*) and percentage change.

## 3 | RESULTS

Complete femoral head ischemia was confirmed in 10/11 piglets as determined by the complete absence of signal enhancement on subtracted CE-MRI. One piglet had partial femoral head ischemia and thus was excluded from the data analysis. In all cases, femoral head ischemia was limited to the epiphysis of the femoral head, whereas the metaphysis was perfused.

**FIGURE 3:**
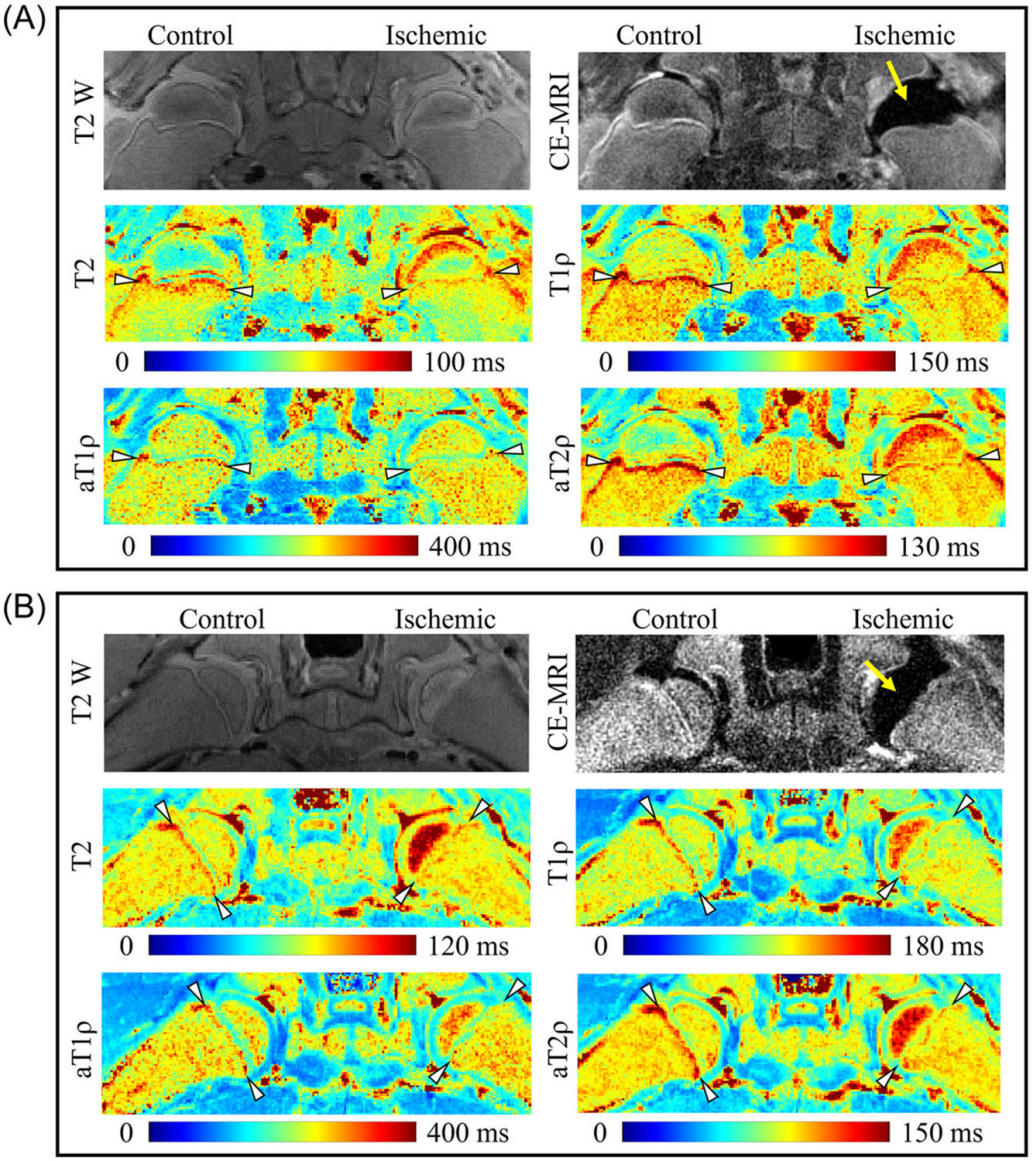
T2-weighted, subtracted CE-MRI, and relaxation time maps of two representative piglets (A and B). Subtracted CE-MRI confirmed complete femoral head ischemia in the epiphysis of the operated hip (yellow arrow). T2, T1ρ, aT1ρ, and aT2ρ relaxation times were decreased in the metaphyseal spongiosa of the ischemic versus control femoral heads (the location of the metaphyseal spongiosa is indicated by the arrowheads).

Relaxation time maps for two representative piglets are shown in Figure 3. Qualitatively, differences in the metaphyseal relaxation times were apparent in the control versus ischemic femoral heads. In the control femoral heads, the secondary spongiosa had considerably longer relaxation times than the primary spongiosa and mature metaphysis (arrowheads). In contrast, in the ischemic femoral heads, the relaxation times in the secondary spongiosa were reduced.

**FIGURE 4:**
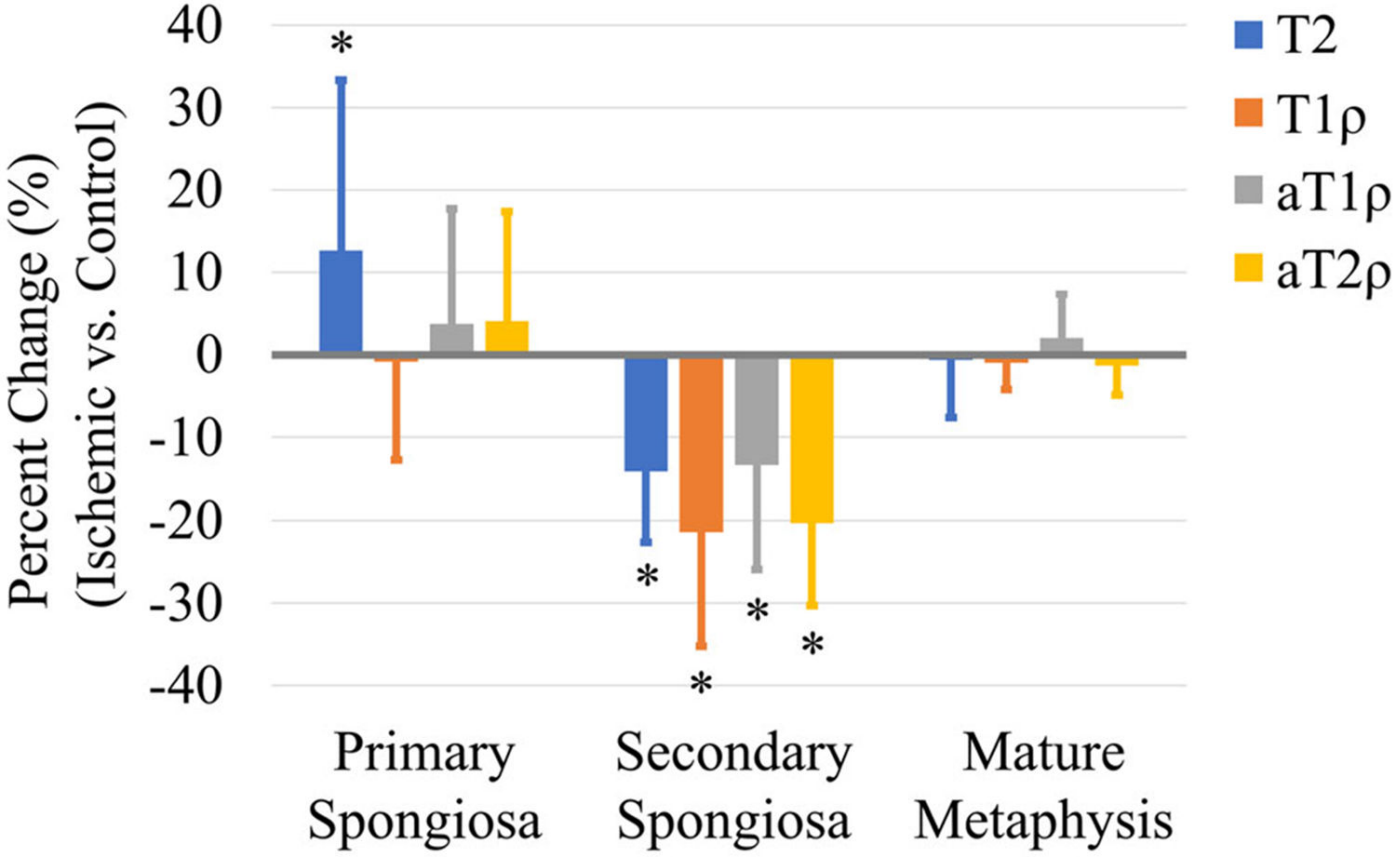
Average percent change in relaxation times in the primary and secondary spongiosa and mature metaphysis of the ischemic versus control femoral heads (*n* = 10 pairs). T2 values increased in the primary spongiosa, and all four relaxation times decreased in the secondary spongiosa of the ischemic versus control femoral heads. No differences were observed in the mature metaphysis. *p < 0.05.

The line profile analyses revealed several differences in the relaxation times of the metaphysis between the ischemic versus control femoral heads (Table 2 and Figure 4). In the primary spongiosa, T2 values were significantly increased in the ischemic versus control femoral heads (*p* = 0.029; *d* = 0.7), with an average percent increase of 13% ± 17%; the other relaxation times did not show a significant difference. In the secondary spongiosa, all four relaxation times were significantly decreased in the ischemic versus control femoral heads: T2 (*p* = 0.013; *d* = 1.4), T1ρ (*p* < 0.001; *d* = 1.4), aT1ρ (*p* = 0.009; *d* = 1.0), and aT2ρ (*p* < 0.001; *d* = 1.8) decreased on average by 14% ± 8%, 21% ± 14%, 13% ± 13%, and 20 ± 10%, respectively. In the mature metaphysis, there were no significant differences in relaxation times in the ischemic versus control femoral heads.

**FIGURE 5:**
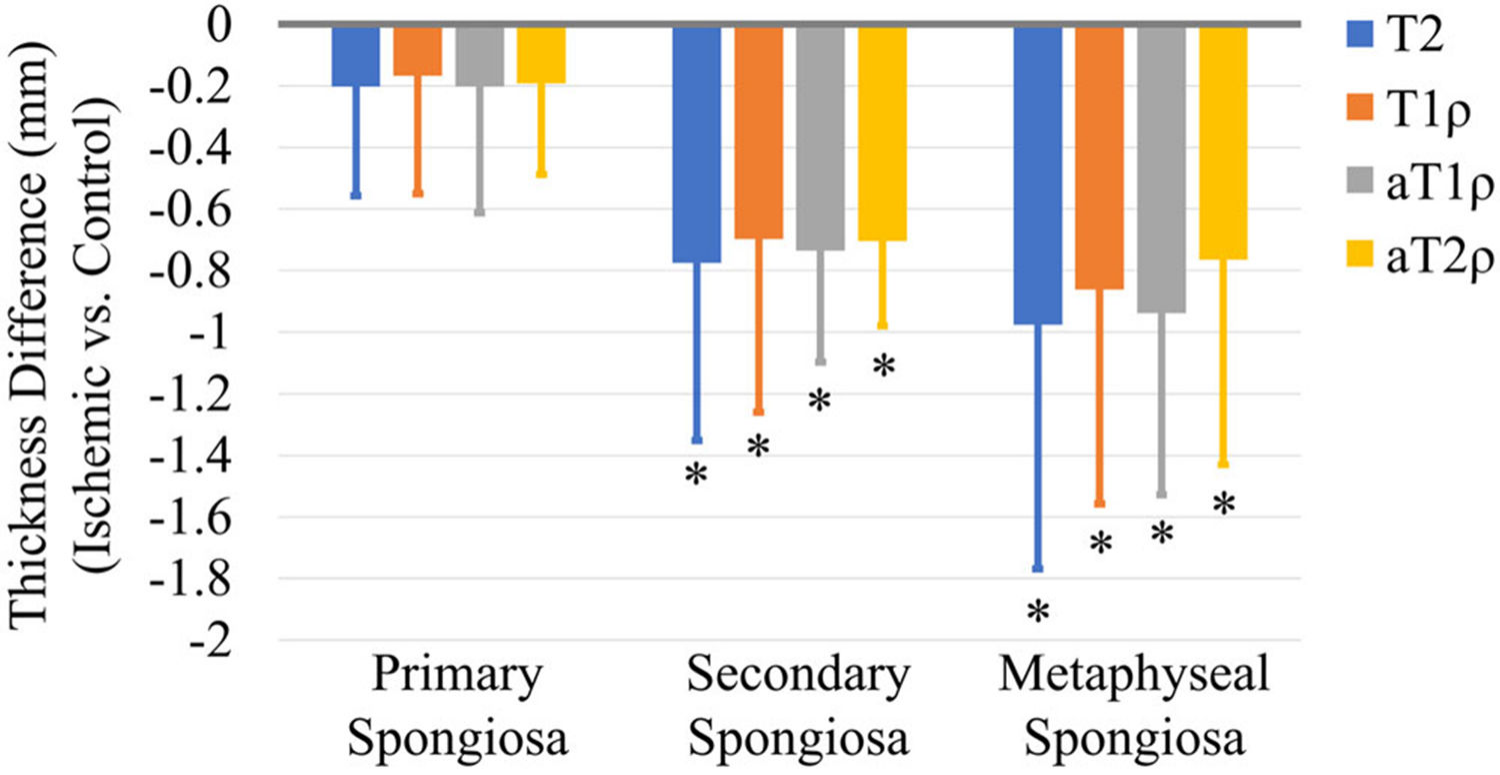
Average difference in primary spongiosa, secondary spongiosa, and total metaphyseal spongiosa thicknesses between the ischemic versus control femoral heads (*n* = 10 pairs). Secondary and total metaphyseal spongiosa thicknesses were significantly decreased in the ischemic femoral heads when measured using all four relaxation time maps. The primary spongiosa thickness also decreased on average, but the difference was not statistically significant. *p < 0.05.

The line profile analyses also identified differences in the thicknesses of the metaphyseal subregions (Table 3 and Figure 5). Secondary spongiosa thickness was significantly decreased in the ischemic versus control femoral heads for each of the relaxation time maps: T2 (*p* = 0.002; *d* = 1.3), T1ρ (*p* = 0.004; *d* = 1.2), aT1ρ (*p* < 0.001; *d* = 2.0), and aT2ρ (*p* < 0.001; *d* = 2.4) maps resulted on average decreases of 38% ± 19%, 35% ± 14%, 37% ± 12%, and 38% ± 13%, respectively. While there was an overall decrease in primary spongiosa thickness in the ischemic versus control femoral heads, these findings were not statistically significant: T2 (*p* = 0.143; *d* = 0.5), T1ρ (*p* = 0.095; *d* = 0.6), aT1ρ (*p* = 0.066; *d* = 0.7), and aT2ρ (*p* = 0.17; *d* = 0.6) maps resulted on average decreases of 15% ± 38%, 11% ± 20%, 13% ± 24%, and 8% ± 28%, respectively. The total metaphyseal spongiosa thickness was significantly decreased in the ischemic versus control femoral heads for each of the relaxation time maps: T2 (*p* = 0.004; *d* = 1.2), T1ρ (*p* = 0.003; *d* = 1.3), aT1ρ (*p* < 0.001; *d* = 2.3), and aT2ρ (*p* < 0.001; *d* = 1.7) maps resulted on average decreases of 29% ± 20%, 26% ± 15%, 28% ± 11%, and 27% ± 16%, respectively.

**FIGURE 6:**
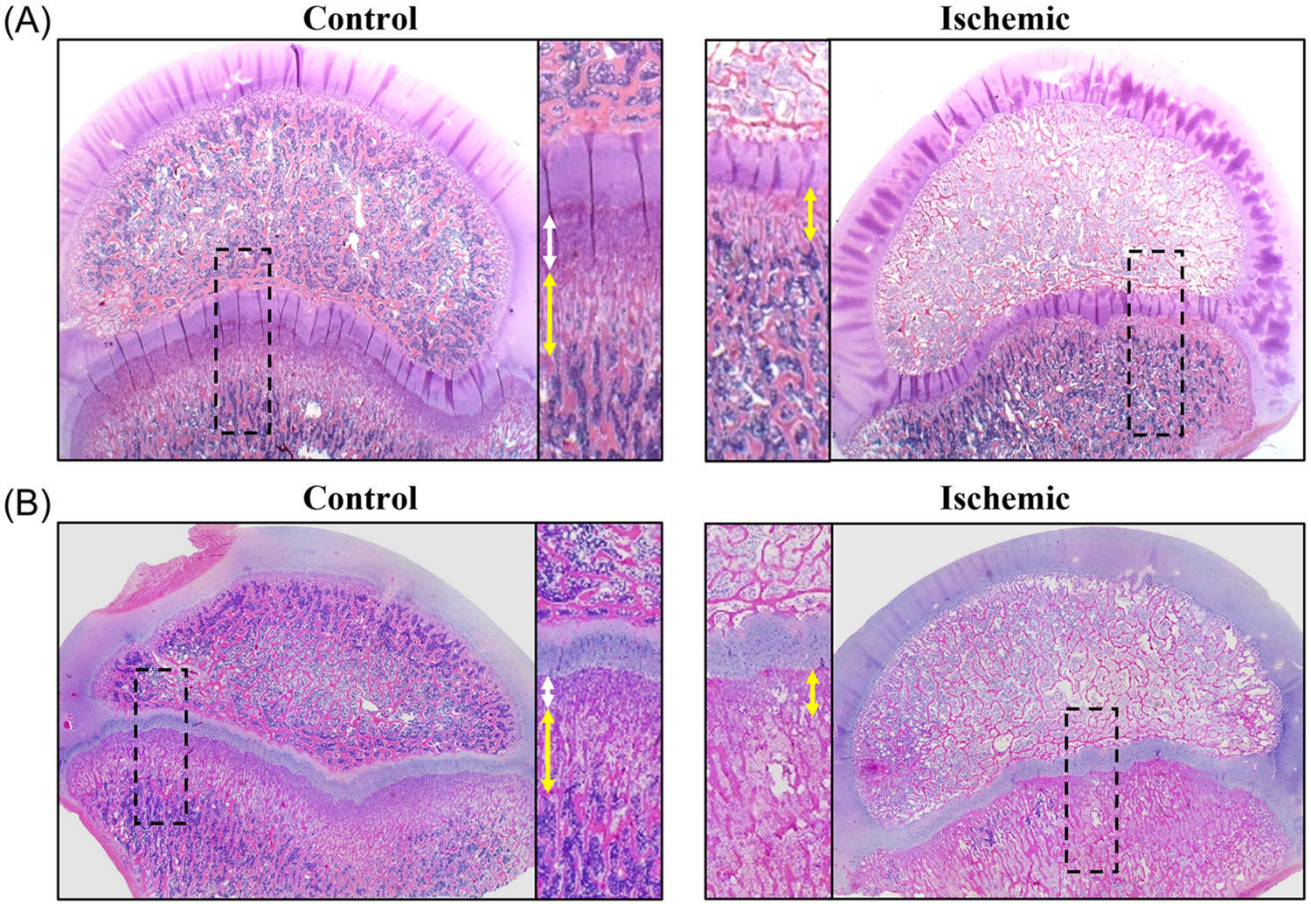
H&E-stained photomicrographs at 0.5× magnification of two pairs of control and ischemic femoral heads (A and B) one week after onset of ischemia in the epiphysis. Magnified views of the boxed regions of the growth plate and metaphysis are also shown adjacent to the corresponding photomicrograph. On the magnified views, arrows indicate the extent of the metaphyseal primary spongiosa (white arrows) and secondary spongiosa (yellow arrows). In the ischemic femoral heads, the primary spongiosa is nearly inapparent, and the secondary spongiosa is thinned with its partial conversion to mature metaphysis.

Results of the qualitative histological analysis identified thinning of the metaphyseal primary and secondary spongiosa in the ischemic versus control femoral heads. Typical findings are shown in Figure 6. In the control femoral heads, the primary spongiosa consisted of dense spicules of predominantly calcified cartilage, and the secondary spongiosa consisted of trabeculae predominated by bone and intermingled with a marrow space with an increased vascular component as compared to the primary spongiosa. In the ischemic femoral heads, the primary spongiosa was thinned to nearly absent, and the secondary spongiosa also was considerably thinned, bringing the more mature, cellular bone marrow and trabecular bone of the mature metaphysis closer to the growth plate.

## 4 | DISCUSSION

Our findings support that T2, T1ρ, aT1ρ, and aT2ρ relaxation time mapping techniques are sensitive in detecting changes to the metaphyseal spongiosa as a result of ischemic injury to the epiphysis of the femoral head. All four relaxation times were sensitive to changes within the secondary spongiosa, and T2 was sensitive to the changes in the primary spongiosa. Although not statistically significant, there was a reduction in primary spongiosa thickness following femoral head ischemia, suggesting either interruption or a reduced rate of primary spongiosa production in the face of continual conversion of primary spongiosa into secondary spongiosa. We also observed a significant decrease in secondary spongiosa thickness, which additionally implies that the secondary spongiosa was converted into more mature metaphyseal bone at a faster rate than it was forming.

The secondary spongiosa had the most prominent metaphyseal differences in the relaxation time maps between the ischemic and control femoral heads. Whereas the normal developing metaphysis of the control femoral heads had longer relaxation times in the secondary spongiosa compared to the mature metaphysis, these relaxation times were significantly decreased in the ischemic femoral heads. On the other hand, the ischemic injury did not affect relaxation times in the mature metaphysis. Additionally, the secondary spongiosa was thinned on the relaxation time maps of the ischemic heads. The secondary spongiosa is a metabolically active region that remodels the immature, woven bone of the calcified primary spongiosa into the trabecular bone and bone marrow of the mature metaphysis. The metabolic demand is supported by increased vascularity in the secondary spongiosa compared to the mature metaphysis.^31^ On histological review, the secondary spongiosa had a relatively low cellularity as compared to the more distal, mature metaphysis in both the control and ischemic femoral heads. Each of these factors likely contributes to relatively long relaxation times in this region: the relaxation times of fluids such as blood are relatively long compared to other tissues such as bone and hematopoietic bone marrow,^32^ so a greater blood volume fraction may lead to an increase in the relaxation times; and the greater extracellular space with fewer hematopoietic cells will increase the amount of free water, which also has increased relaxation times.^33^ Collectively, our findings suggest that, in the case of complete ischemia of the femoral epiphysis, the growth of the metaphysis was slowed or halted, leading to thinning of the secondary spongiosa as well as a gradual change in composition throughout its thickness as its maturation outpaced its new formation. As a result, T2 and T1ρ relaxation times (and the aT1ρ and aT2ρ variants) decreased as the composition of the secondary spongiosa became more like that of the mature metaphysis.

Although not statistically significant, we also observed differences in the primary spongiosa consistent with its thinning in the ischemic versus control femoral heads using relaxation time mapping. We found that the primary spongiosa thickness was decreased on average by approximately 0.2 mm on the relaxation time maps in the ischemic femoral heads. This is consistent with a prior histological study of the growth plate in the LCPD piglet model, which measured an average decrease in the thickness of the primary spongiosa of 0.31 mm 1 week after onset of ischemia.^18^ However, since our relaxation time maps had a nominal spatial resolution of 0.52 mm, and with the effects of partial volume averaging, the ability to resolve these small differences with MRI are limited and thus likely explains the lack of statistical significance. The thinning of the primary spongiosa is likely driven by two processes following ischemic injury to the epiphysis: first, arrest of the growth plate limits its ability to generate new primary spongiosa; and second, continued conversion of the distal portion of the primary spongiosa into secondary spongiosa. These processes may also have contributed to the significant increase in T2 relaxation times in the primary spongiosa in the ischemic femoral heads by limiting the formation of new calcification and/or causing early breakdown to the proximal portion of the primary spongiosa.

Our findings in the LCPD piglet model support that relaxation time mapping may provide a noninvasive means to detect changes in the metaphysis. If these findings prove to be clinically translatable, then they can potentially help inform clinical management of children with LCPD. T2 relaxation time mapping is a clinically available technique that may be useful to probe changes in both the primary and secondary spongiosa. T1ρ and aT2ρ relaxation time mapping may provide additional sensitivity in detecting changes in the secondary spongiosa; of the four relaxation time mapping techniques we investigated, these techniques had the greatest effect size in this region. Relaxation time mapping techniques can also provide higher spatial resolution and potentially complementary information to diffusion-weighted imaging, which is another quantitative MRI technique that can detect changes in the metaphysis in LCPD.^9-14^ The metaphyseal diffusion changes observed in other studies do not have a confirmed source, but they may relate to a repair response in the later stages of LCPD such as increased perfusion, which may be a consequence of the early-stage injury and growth disruption we observed in this study. Specifically, it is possible that the early changes in maturation of the metaphyseal spongiosa we observed are associated with subsequent vascular invasion of the growth plate resulting in transphyseal revascularization of the ischemic epiphysis, which is generally thought to lead to worse clinical outcomes in LCPD than revascularization through the epiphyseal cartilage overlying the femoral epiphysis.^10^ Thus, relaxation time changes in the metaphysis may be an early predictor of clinical outcome in patients with LCPD.

Our study has several limitations. We evaluated the piglets at only one time point corresponding to the early, avascular stage of LCPD. While our sample was sufficient to detect significant differences in metaphyseal relaxation times, our findings motivate further investigation of these changes at later time points to determine whether they contribute to additional injury and/or repair of the proximal femur. Our analysis was also limited to bilateral 2D relaxation time mapping of a single imaging slice, which may result in some degree of spatial misregistration between the ischemic and control femoral heads. While 2D relaxation time mapping was selected to provide relatively high spatial resolution and signal-to-noise ratio for this study, ideally 3D relaxation time mapping would be acquired to reduce potential differences based on slice positioning. Furthermore, in future studies, longitudinal 3D measurements would allow changes in the metaphysis to be evaluated in the ischemic femoral head throughout disease progression. This could include imaging the affected femoral head preoperatively to avoid any potential variation between the operated and contralateral-control femoral heads due to either normal growth or effects of the surgery on the contralateral limb (e.g., due to changes in loading). Furthermore, 3D measurements would potentially allow for spatial co-registration of the MRI images and histological photomicrographs for further validation of the MRI thickness measurements and underlying compositional changes.

In conclusion, T2, T1ρ, aT1ρ, and aT2ρ relaxation time mapping techniques can detect early changes in the metaphysis following ischemic injury to the epiphysis of the femoral head in a piglet model of LCPD. These techniques may be clinically useful to improve our understanding of the pathogenesis of LCPD and to predict clinical outcome to inform treatment decisions.

## Figures and Tables

**TABLE 1:** 3 T MRI imaging parameters.

	T2, T1ρ, aT1ρ, and aT2ρ mapping	Subtracted CE-MRI
Sequence	Magnetization-prepared 2D TSE	2D TSE
Field of view (mm)	200 × 200	200 × 170
Sampling matrix	384 × 384	448 × 380
In-plane resolution (mm^2^)	0.52 × 0.52	0.45 × 0.45
Slices/thickness (mm)	1/2.0	20/2.0
TR/TE (ms)	2220/13	970/21
Flip angle (degrees)	180	150
Bandwidth (Hz/px)	130	260
Fat sat	No	Yes
GRAPPA	No	R = 2
Turbo factor	8	4
T2 and T1ρ Prep times (ms)	0, 20, 40, 60, 80	–
aT1ρ and aT2ρ Prep times (ms)	0, 24, 48, 72, 96	–
T1ρ spin-lock frequency (Hz)	350	–
aT1ρ and aT2ρ spin-lock pulse	HS1, 6ms duration, B1_max_ = 1000 Hz	–
Scan time	9:24 (×4 maps)	3:10 (×2 repetitions)

**TABLE 2:** Relaxation time differences in the primary spongiosa, secondary spongiosa, and mature metaphysis of the ischemic versus control femoral heads across *n* = 10 piglets.

		Control (ms)	Ischemic (ms)	Paired difference (ms)	95% CI	*t*	*p*-Value	Effect size (Cohen's *d*)	Percent change (%)
**Primary spongiosa**	T2	57.9 ± 5.4	64.6 ± 7.1	6.7 ± 9.8	[0.8, 12.6]	2.4	**0.029** *	0.7	13 ± 17
	T1ρ	87.3 ± 2.9	86.5 ± 9.8	−1 ± 10	[−7.9, 6.3]	0.2	0.81	0.1	−1 ± 12
	aT1ρ	176.8 ± 6.6	183 ± 23	6 ± 25	[−10, 22]	0.8	0.42	0.2	4 ± 14
	aT2ρ	75.1 ± 2.5	78.0 ± 9.0	2.9 ± 9.8	[−3.9, 9.2]	1.0	0.34	0.3	4 ± 13
**Secondary spongiosa**	T2	92 ± 12	78.5 ± 9.9	−13.3 ± 9.3	[−23.4, −3.2]	2.8	**0.013** *	1.4	−14 ± 8
	T1ρ	145 ± 16	113 ± 19	−32 ± 23	[−48, −15]	4.0	**<0.001** *	1.4	−21 ± 14
	aT1ρ	300 ± 37	258 ± 28	−43 ± 41	[−74, −12]	2.9	**0.009** *	1.0	−13 ± 13
	aT2ρ	125 ± 15	99 ± 14	−26 ± 14	[−39, −13]	4.1	**<0.001** *	1.8	−20 ± 10
**Mature metaphysis**	T2	65.4 ± 7.0	64.9 ± 7.2	−0.5 ± 4.0	−7.2, 6.2]	0.2	0.88	0.1	−1 ± 7
	T1ρ	103.1 ± 7.2	102.3 ± 7.7	−0.8 ± 3.8	[−7.8, 6.2]	0.2	0.81	0.2	−1 ± 4
	aT1ρ	245 ± 15	249 ± 14	5 ± 12	[−9, 18]	0.7	0.49	0.4	2 ± 5
	aT2ρ	88.0 ± 8.8	86.9 ± 9.5	−1.1 ± 2.8	[−9.7, 7.5]	0.3	0.79	0.4	−1 ± 4

*Note:* Values are reported as mean ± standard deviation.

* Statistically significant (*p* < 0.05) are in bold.

**TABLE 3:** Differences in primary, secondary, and total metaphyseal spongiosis thickness in the ischemic versus control femoral heads across *n* = 10 piglets.

		Control (mm)	Ischemic (mm)	Paired difference (mm)	95% CI	*t*	*p*-Value	Effect size (Cohen's *d*)	Percent change (%)
**Primary Spongiosa Thickness**	T2	1.12 ± 0.22	0.91 ± 0.31	−0.21 ± 0.40	[−0.49, 0.08]	1.6	0.143	0.5	−15 ± 38
	T1ρ	1.25 ± 0.33	1.08 ± 0.27	−0.17 ± 0.29	[−0.37, 0.04]	1.9	0.095	0.6	−11 ± 20
	aT1ρ	1.35 ± 0.40	1.15 ± 0.30	−0.21 ± 0.31	[−0.43, 0.02]	2.1	0.066	0.7	−13 ± 24
	aT2ρ	1.32 ± 0.49	1.14 ± 0.30	−0.18 ± 0.38	[−0.45, 0.09]	1.5	0.17	0.6	−8 ± 28
**Secondary Spongiosa Thickness**	T2	1.90 ± 0.60	1.13 ± 0.31	−0.77 ± 0.58	[−1.19, −0.36]	4.2	**0.002** *	1.3	−38 ± 19
	T1ρ	1.80 ± 0.78	1.11 ± 0.33	−0.70 ± 0.56	[−1.10, −0.29]	3.9	**0.004** *	1.2	−35 ± 14
	aT1ρ	1.92 ± 0.46	1.18 ± 0.25	−0.74 ± 0.36	[−0.99, −0.48]	6.4	**<0.001** *	2.0	−37 ± 12
	aT2ρ	1.78 ± 0.31	1.11 ± 0.30	−0.68 ± 0.29	[−0.89, −0.48]	7.5	**<0.001** *	2.4	−38 ± 13
**Total Metaphyseal Spongiosa Thickness**	T2	3.02 ± 0.75	2.04 ± 0.36	−0.98 ± 0.81	[−1.56, −0.40]	3.8	**0.004** *	1.2	−29 ± 20
	T1ρ	3.05 ± 0.87	2.18 ± 0.43	−0.87 ± 0.68	[−1.35, −0.38]	4.1	**0.003** *	1.3	−26 ± 15
	aT1ρ	3.27 ± 0.54	2.33 ± 0.40	−0.94 ± 0.41	[−1.24, −0.65]	7.3	**<0.001** *	2.3	−28 ± 11
	aT2ρ	3.11 ± 0.57	2.25 ± 0.49	−0.86 ± 0.51	[−1.22, −0.50]	5.4	**<0.001** *	1.7	−27 ± 16

*Note:* Values are reported as mean ± standard deviation.

* Statistically significant (*p* < 0.05) are in bold.
